# A cross-over, randomised feasibility study of digitally printed versus hand-painted artificial eyes in adults: PERSONAL-EYE-S - a study protocol [version 2; peer review: 2 approved]

**DOI:** 10.3310/nihropenres.13311.1

**Published:** 2023-03-15

**Authors:** Taras Gout, Tim Zoltie, Amie Woodward, Elizabeth Coleman, Florien Boele, Bernard Chang, Paul Bartlett, Sarah Ronaldson, George Kalantzis, Mike Theaker, Nabil El-Hindy, Emma Walshaw, Judith Watson

**Affiliations:** 1 Leeds Artificial Eye Service, Leeds Dental Institute, Leeds Teaching Hospitals NHS Trust, Worsley Building, Leeds, LS2 9LU, UK; 2 School of Dentistry, Faculty of Medicine and Health, University of Leeds, Worsley Building, Leeds, LS2 9LU, UK; 3 York Trials Unit, Department of Health Sciences, University of York, York, YO10 5DD, UK; 4 Leeds Institute of Health Sciences, Faculty of Medicine and Health, University of Leeds,, Leeds, LS2 9JT, UK; 5 Leeds Institute of Medical Research at St James’s, St James’s University Hospital, University of Leeds, Leeds, LS9 7TF, UK; 6 Department of Ophthalmology, Leeds Teaching Hospitals NHS Trust, Leeds, LS9 7TF, UK; 7 Patient and Public Involvement representative, Leeds, UK

**Keywords:** artificial eyes, feasibility, cross-over, qualitative, quality of life, health economics

## Abstract

**Background/objectives:**

Around 11,500 artificial eyes are required yearly for new and existing patients. Artificial eyes have been manufactured and hand-painted at the National Artificial Eye Service (NAES) since 1948, in conjunction with approximately 30 local artificial eye services throughout the country. With the current scale of demand, services are under significant pressure. Manufacturing delays as well as necessary repainting to obtain adequate colour matching, may severely impact a patient’s rehabilitation pathway to a normal home, social and work life. However, advances in technology mean alternatives are now possible. The aim of this study is to establish the feasibility of conducting a large-scale study of the effectiveness and cost-effectiveness of digitally printed artificial eyes compared to hand-painted eyes.

**Methods:**

A cross-over, randomised feasibility study evaluating a digitally-printed artificial eye with a hand-painted eye, in patients aged ≥18 years with a current artificial eye. Participants will be identified in clinic, via ophthalmology clinic databases and two charity websites. Qualitative interviews will be conducted in the later phases of the study and focus on opinions on trial procedures, the different artificial eyes, delivery times, and patient satisfaction.

**Discussion:**

Findings will inform the feasibility, and design, of a larger fully powered randomised controlled trial. The long-term aim is to create a more life-like artificial eye in order to improve patients' initial rehabilitation pathway, long term quality of life, and service experience. This will allow the transition of research findings into benefit to patients locally in the short term and National Health Service wide in the medium to long term._

**ISRCTN registration:**

ISRCTN85921622 (prospectively registered on 17/06/2021)

## Introduction

The National Artificial Eye Service in England, UK has over 48,000 patients in its database alone and around 1,417 patients have surgery to remove their eye each year^1^. Replacement artificial eyes are made every 2-6 years for adults, with around 11,500 artificial eyes required yearly for new and existing patients. These eyes are manufactured by the National Artificial Eye Service (NAES) in conjunction with around 30 local artificial eye services. With such a level of demand, services are under increasing pressure which can result in delays in provision of artificial eyes.

Following an operation to remove a diseased eye, such as for eye cancer, a well fitted, life-like artificial eye can help rehabilitation. However, many patients suffer from anxiety and depression associated with their perceived disfigurement^2–4^, although the impact on Health-Related Quality of Life (HRQoL) is unknown.

The NAES has hand-painted artificial eyes since its integration into the National Health Service (NHS) in 1948. Local orbital prosthetists see patients in clinics, and use a non-standardised set of artificial eyes to colour match the appearance of the unaffected eye. A centralised site of laboratory technicians then manufacture the eye in six to ten weeks. However, achieving a good colour match is difficult and often requires multiple revisions.

This issue has been highlighted by Patient and Public Involvement (PPI) representatives as causing distress and delaying rehabilitation: many reported waiting up to 1 year for a good match. Family members or friends (hereafter called close contacts) are also impacted by the quality of artificial eyes as ill-matching eyes can cause distress. Digital photography for colour matching only the iris, and not the rest of the eye, have been described previously^5^.

These issues prompted the novel design and development of digitally-printed artificial eyes by Leeds Artificial Eye Service (LAES), Leeds, UK. Early work demonstrated that patients can receive a more life-like match, often within 2 weeks and requiring fewer multidisciplinary clinic visits.

Further research has to determine whether digitally printed eyes result in improvements in patients’ HRQoL and satisfaction. A large-scale randomised controlled trial (RCT) is needed to evaluate the effectiveness and cost-effectiveness of digitally-printed compared to hand-painted artificial eyes in order to inform clinical practice. First, we will undertake a feasibility RCT to see if it is possible to conduct a larger study.

## Study aims and objectives

The primary aim of this study is to determine the feasibility of conducting a RCT of the effectiveness and cost-effectiveness of digitally-printed artificial eyes compared to hand-painted eyes.

The specific objectives are to:

- Determine the eligibility rate;
- Determine patient recruitment rates;
- Identify retention rates, data fidelity and missing data;
- Identify a primary outcome measure(s) for a future trial (if feasibility established – by considering the amount of missing data, and any ceiling/floor effects seen within the measures);
- Test study procedures and data collection tools and management;
- Establish scalability of the current service.

The feasibility of the future trial will be assessed based on whether:

- Patient recruitment and retention rates indicate recruitment for a full-scale RCT is plausible;
- Outcome measures and fidelity evaluation data are successfully collected. Measures with over 10% missing data may be modified/replaced prior to the main trial;
- Qualitative data confirms willingness of patients to be recruited, randomised and find research processes acceptable; and healthcare professionals’ opinions on the different artificial eyes, views on delivery times and patient satisfaction proves acceptable.

## Subjects and methods

### Study design

The study is a single centre, cross-over, randomised controlled, open feasibility study. Eligible patients who provide written informed consent will be randomised to:

A: receive a digitally-printed artificial eye first (intervention), followed by a hand-painted artificial eye (control); or
B: receive a hand-painted artificial eye first, followed by a digitally-printed artificial eye.

**Figure 1:**
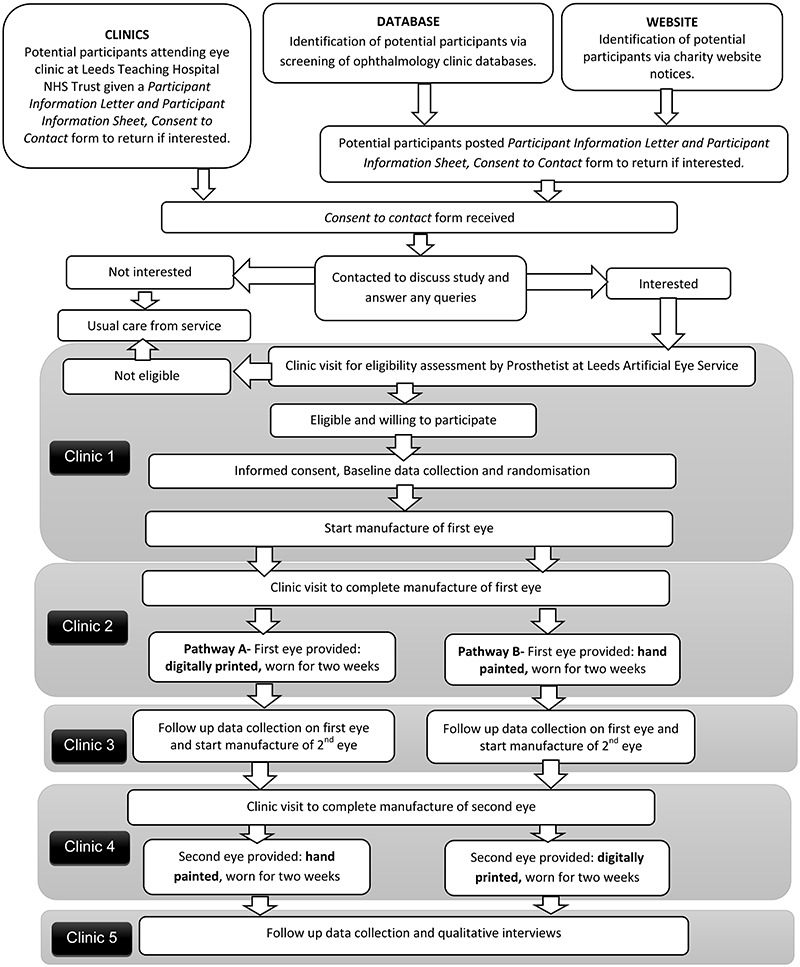
Summary of patient/participant pathway.

A flow diagram demonstrating participant pathway through the study is provided in Figure 1. Participants will be asked to attend five clinics approximately 2 weeks apart. Clinic 1- final eligibility, consent and baseline data; Clinic 2 – fitting of first eye; Clinic 3 – follow-up data on first eye; Clinic 4 - fitting of second eye; Clinic 5 – follow-up data on second eye and qualitative interviews. Between Clinics 3 and 4, participants can either continue to wear the first allocated eye, or revert to their original artificial eye. No concomitant care or interventions are prohibited (with the exception of participation in other artificial eye studies) and participants are allowed to keep their original artificial eye during the study.

All study documents including consent forms and data collection forms can be found as *Extended data*^6^.

### Setting

LAES at Leeds Teaching Hospitals NHS Trust will conduct participant identification, assess eligibility, consent participants and deliver the interventions.

Artificial eyes are classed by the Medicines and Healthcare products Regulatory Agency (MHRA) as “custom-made medical devices” intended for the sole use of a particular patient. Products manufactured in-house in a healthcare establishment that is conducting the study are not subject to the provisions of UK Medical Devices Regulations 2002, provided that the device is being manufactured and used on patients within the sole legal entity. There is no objective/intention to place the device on the market, and we manufacture the device inhouse for our own patients within Leeds Teaching Hospitals NHS Trust and therefore in the context of this study, does not constitute a medical devices trial requiring MHRA notification.

### Study population

#### Inclusion criteria

- aged ≥ 18 years old;
- longstanding artificial eye user (≥12 months post-operation);
- requires a replacement artificial eye;
- able to complete the English language outcome measures (independently or with assistance).

#### Exclusion criteria

- ongoing clinical concerns with respect to their artificial eye use (e.g. poor eye socket healing, extrusion, dehiscence);
- has bilateral artificial eyes;
- pregnant women or persons currently shielding (due to ongoing pandemic, to avoid unnecessary visits to clinic);
- currently participating in another study evaluating their artificial eye;
- unable or unwillingness to provide written informed consent.

#### Recruitment

Prospective participants will be identified via three main methods: in clinic, via database screening, or via notification placed on the Royal National Institute of Blind People and Blind Veterans UK websites.

Patients will be handed or posted a study invitation pack containing a Patient Invitation Letter, Participant Information Sheet, a Consent to Contact form, information pack for a close contact, and a pre-paid envelope. Patients will be asked to return the Consent to Contact form to York Trials Unit (YTU) if they wish to be contacted about the trial. Upon receipt of a patient’s Consent to Contact form, YTU will contact them to discuss any queries. The contact details of patients who are willing to be assessed for eligibility will be passed to LAES to arrange an appointment.

#### Eligibility and consent

Interested patients will be given an appointment to attend clinic for a final eligibility assessment.

Interested patients will be asked to approach a close contact (e.g. friend, family member, spouse), who might be willing to participate alongside the patient, in which case a specific information pack will be provided. Patients can take part without a close contact, but not vice versa.

If eligible and willing, written informed consent will be obtained from patients and, if applicable, their close contacts according to Good Clinical Practice guidelines.

#### Baseline assessment

Following consent procedures, baseline data will be collected according to the schedule in Table 1.

Eye history and assessment will include past medical history; past ocular and artificial eye history; current medications; and artificial eye socket examination (general socket health, fit and comfort of artificial eye, any current problems).

#### Randomisation

Following consent and baseline data collection (to ensure allocation concealment), an authorised team member at site will access a secure internet-based service hosted by the UKCRC-accredited YTU to obtain the order of the participant’s treatment allocation. Participants will be randomised on a 1:1 basis using block randomisation. Block sizes will not be disclosed, to ensure concealment. No stratification will be used.

### Level of masking

By the nature of the study treatments, masking of participants and clinic staff is not possible and procedure for un-masking is not necessary.

### Study treatments

All participants will have an eye socket impression taken for a fitted artificial eye. The artificial eye shape will be made by the LAES maxillofacial laboratory.

#### Digitally printed artificial eye (Intervention)

Digital photos of the unaffected eye will be printed onto specialised adhesive paper pressed onto the artificial eye.

#### Hand-painted artificial eye (Control)

Colour matching the unaffected eye will be done by the imaging department using a colour guide. The artificial eye shape and colour guide number are then sent to iProsthetics (Norwich, UK), to hand-paint and encapsulate with acrylic the artificial eye.

Each eye will be worn for at least two weeks. Attendance at clinics will be facilitated by participants having any travel expenses for additional protocol visits reimbursed. It is hoped this will increase adherence to the protocol in terms of clinic visits. Participants will be able to retain both new artificial eyes following study completion.

### Data collection

A summary of all data collection is presented in Table 1.

### Feasibility data

Data collected relating to recruitment will be: number of eligible patients, and the proportion of those that were screened: number approached for consent and not approached (and reason why); number approached who provide consent and do not provide consent (and reasons why). In addition, proportion of randomised participants withdrawing (with reasons) and lost to follow-up will be collected (and reason why, where possible).

### Staff-collected data and preference questionnaire

Staff will collect data at baseline, Clinic 3 and Clinic 5 which will include information on eye socket health and adverse events (AEs). Data will also be collected at each clinic on tasks undertaken (including manufacturing steps both eyes) and time taken.

Following completion of the study by all participants, the healthcare professionals will also be asked to view eye-band images comparing both eyes, stating which they prefer.

### Participant self-reported data

At baseline, Clinic 3 and Clinic 5 the following participant self-reported data will be collected:

- Short Form 36 (SF-36): a generic HRQoL measure^7^.
- EQ-5D-5L (EuroQol-5 dimensions-5 levels): a generic HRQoL measure^8^.
- Vision Quality of Life Index (VisQol): assesses impact of vision loss^9^.
- Connor-Davidson Resilience Scale-10 (CD-RISC-10): provides a measure of resilience^10^.
- Derriford Appearance Scale short form (DAS24): assesses distress and difficulties in living with disfigurement and problems of appearance^11^.
- Participant satisfaction: a brief study-specific questionnaire to assess participant satisfaction using a 0–10 scale. Eye-band image selection comparing both eyes will also be used.
- Resource use: a study-specific questionnaire on hospital attendances, admissions, and primary care visits (e.g. GP, nurse), in relation to their artificial eye.

### Qualitative data collection

Qualitative interviews will be done in the later phases of the study and focus on opinions on trial procedures, the different artificial eyes, delivery times, and patient satisfaction. The study consent form will also contain statements in relation to consent to interview for this qualitative aspect of the study. Re-confirmation of consent will be taken verbally prior to commencement of the interview. A subset of participants (approximately N=15), and their close contacts (approximately N=15) will be interviewed after both artificial eyes have been trialled. Interviews, lasting ~45 minutes, can be done face-to-face at a location of the person’s preference, over the phone, or via video-call and will be audio-recorded. Where possible, individual interviews will be conducted to allow each member of the dyad to freely express their feelings. Data collection will continue until saturation is achieved^12^.

Approximately 5 members of the manufacture, ophthalmology, maxillofacial, and prosthetists teams will be invited to take part in a semi-structured interview lasting ~20 minutes. Following consent, interviews will take place face-to-face, over the phone or via videocall.

### Close contact data

Close contacts will be asked to complete a study-specific satisfaction questionnaire after their friend/relative has worn each eye (Clinics 3 and 5) and asked to compare photographs of both eyes stating a preference (Clinic 5).

### Data management

Data collected as part of this research includes questionnaires, clinical assessments, and qualitative data from interviews. Data will be collected through designed questionnaires on paper which will be scanned using specialist software. The data will be checked against the hard copy of the questionnaire, error checked and then validation checks run against the database. Queries will be raised with the site if discrepancies are identified during validation. A range of centralised monitoring activities (e.g., eligibility, consent, safety checks) will be undertaken as well as regular site visits to discuss any issues being encountered.

### Attendance, follow-up and withdrawal

Appointments will be offered approximately 2–3 weeks apart. If a participant is unable to attend a scheduled appointment, they will be offered up to two further appointments within a 6-week period. They must attend Clinics 2 and 4 (the study eye fitting appointments) or will be withdrawn from the trial.

If the participant does not attend Clinics 3 or 5, they and their close contact will be mailed the follow-up questionnaire(s) by YTU. If not returned, they will receive a reminder letter followed by a telephone call.

Participants are free to withdraw from the study at any time and with no obligation to provide a reason. A participant may be withdrawn for clinical reasons (e.g. problems related to eye socket, deterioration in health, distress related to loss of eye etc). If a participant fully withdraws (i.e. withdraws from interventions, questionnaires and interview), their close contact is also withdrawn, but not vice versa.

### Storage and confidentiality of data and patient records

Data will be collected through designed questionnaires identified by a unique participant trial number only. The research teams will hold data according to the General Data Protection Regulation (May 2018). Participant details will be stored on a secure password-protected server located at the University of York. Documents will be stored safely in confidential conditions, either physically or electronically and identifiable data will not be stored with questionnaire data. Qualitative interviews sound files and transcripts will be transferred onto a secure server and the data removed from the portable recording device. Data will be archived by the University of York for a minimum period of 5 years following the end of the study.

### Adverse events

Adverse events (AEs) and serious AEs (SAEs) will be recorded. Intensity and relationship to the study intervention will be described. Examples of AEs related to participation in this study include: infection, inflammation and irritation, pain/discomfort, prosthesis retention, and implant retention.

Ongoing review of AEs will take place during monthly trial management group (TMG) meetings, discussed with the patient advisory group (PAG) and trial steering committee (TSC) and reported to the sponsor and research ethics committee in line with their guidelines.

### Sample size

As this is a feasibility study the primary outcome measure does not inform a sample size calculation. One of the study’s aims is to estimate the within-subject standard deviation for each outcome measure, in order to inform the sample size calculation for a future study. Pilot and feasibility trial literature recommends a sample size of between 24 and 70 to inform reliable estimation of standard deviations^13,14^. Thus, we aim to recruit 35 participants, which, assuming a 15% attrition rate, would allow for 30 participants in the final analysis.

### Analyses

A statistical analysis plan detailing intended analyses will be drafted before the completion of data collection. The trial will be reported according to the CONSORT guidelines, including the extension to randomised cross-over trials^15–17^.

No interim analyses will be conducted. As this is a feasibility trial, no formal stopping rules are in place. At study end, the number of patients screened, eligible, consented and randomised will be summarised. Baseline and outcome data, along with questionnaire return rates and adverse event data, will be summarised overall and by randomised group, and treatment period as appropriate. Within-subject differences for each outcome will also be summarised. To evaluate between-group change in outcome and process variables we will calculate the mean and 95% confidence intervals at baseline, after trialing the first artificial eye, and after trialing the second artificial eye. We will report the number that receive each eye, by group. The number of participants who have a close contact will also be detailed, alongside the return rates for the close contact questionnaires, and associated outcomes.

Continuous variables will be summarised descriptively (mean, standard deviation, median, minimum and maximum) and categorical data will be summarised as counts and percentages. As this is a feasibility trial, statistical hypothesis testing for effectiveness will not be carried out.

The feasibility of undertaking an economic evaluation will be explored; relevant economic data will be identified and corresponding data collection methods investigated. Health outcomes (EQ-5D-5L), healthcare resource use, and costs for patients using the two artificial eye services will be evaluated using descriptive statistics similarly to those described above. Unit costs will be obtained from established costing sources^18,19^, taking an NHS perspective. The cost of the eye services will be estimated to indicate the likely resource implications and corresponding costs. A full economic evaluation will not be conducted, though the health economics work, including exploration of missing economic data, will help guide the cost-effectiveness methods used in a full-scale trial.

Transcribed data from the qualitative interviews will be downloaded into NVivo software package, coded and analysed inductively using thematic content analysis^20,21^. The researcher conducting the interviews will develop the initial coding framework of themes and subthemes. A second researcher will check a sample of data transcripts against the audio recordings, for accuracy, and will interrogate the validity of the coding against the raw data. The coding framework will be developed iteratively throughout the analysis process, through regular discussions leading to consensus. Detailed thematic summaries will be created using a flexible template that covers the research objectives, while allowing for the emergence of further themes.

### Ethical and oversight arrangements

The TMG will monitor the day-to-day management of the trial. Due to the low risk nature of this trial, one independent steering and monitoring committee will set up to undertake the roles traditionally undertaken by the Trial Steering Committee (TSC) and Data Monitoring Committee. Ethical approval was granted by North West – Haydock on 09^th^ June 2021 (Reference 21/NW/0150). The trial has been registered on ISRCTN (registration number: ISRCTN85921622) on 17^th^ June 2021. No protocol modifications have been made since ethical approval was obtained (Protocol v2.0_21.05.2021).

### Patient and Public Involvement (PPI)

A PAG will contribute to study reports and disseminate findings at appropriate events.

The PAG will include at least four patient members and family members who will meet at critical time-points of the study (approximately every 4 months). Their role will include: assisting with the development of the satisfaction questionnaire; identifying barriers/facilitators to participation; identifying appropriate pathways for dissemination; contribute to the writing of the results lay summary; and, assisting with dissemination events. At least one member of the PAG will sit on the TMG.

### Dissemination and projected outputs

Results will be written up and submitted to peer-reviewed journals and presented at relevant national/international ophthalmology conferences. Study findings will be made available to participants, PAG, NHS commissioners and reported on relevant patient-focused websites.

### Study status

Data collection, eye manufacturing and clinic attendance are ongoing.

## Conclusion

The aim of this study is to establish the feasibility of conducting a large-scale study of the effectiveness and cost-effectiveness of digitally-printed artificial eyes compared to hand-painted eyes. The impact will ultimately be realised by a larger fully powered randomised controlled trial, which this feasibility study will inform. The long-term aim is to create a more life-like artificial eye to improve patients’ initial rehabilitation pathway, long term quality of life, and service experience. This will allow the transition of research findings into benefit to patients locally in the short term and NHS wide in the medium to long term.

## Figures and Tables

**Table 1:** Baseline and follow-up data collection.

Timepoint Data	Baseline data (Clinic 1)	After first eye (Clinic 3)	After second eye (Clinic 5)
**Eligibility screening form**	**X**		
**Consent**	**X**		
**Patient reported:**			
Demographics	**X**		
SF-36	**X**	**X**	**X**
VisQoL	**X**	**X**	**X**
EQ-5D-5L	**X**	**X**	**X**
CD-RISC-10	**X**	**X**	**X**
DAS-24	**X**	**X**	**X**
Satisfaction		**X**	**X**
Health care resource use	**X**	**X**	**X**
Eye band image preference			**X**
**Clinician collected data**			
Medical history	**X**		
Eye socket health and artificial eye check	**X**	**X**	**X**
Adverse events		**X**	**X**
**Close contact data:**			
Satisfaction questionnaire		**X**	**X**
Eye band image preference			**X**
**Semi-structured interviews with participants**			**X**
**Semi-structured interviews with close contact**			**X**
**Semi-structured interviews with healthcare professionals**			**X**
Healthcare professionals- eye band image preference			**X**

## Data Availability

No data are associated with this article. Following completion of the study the anonymised data will be shared with any person who makes a reasonable request in writing to the York Trials Unit. Qualitative data will be retained at the University of Leeds. Open Science Framework: PERSONAL-EYEs. https://doi.org/10.17605/OSF.IO/GWXUN^6^. This project contains the following extended data: - Consent forms - Consent to Contact Forms - Data Collection forms - Participant Information Sheets - Protocol Data are available under the terms of the Creative Commons Attribution 4.0 International license (CC-BY 4.0).

## Plain english summary

### Aims of the research

A large-scale study is needed to see how traditional hand-painted artificial eyes compare with digitally-printed eyes in terms of patient satisfaction and cost. Before that, this smaller study is needed to show whether people would take part, are happy to provide health information, and learn what they liked and did not like about taking part.

### Background to the research

After operations to remove a diseased or blind, painful eye many patients suffer from anxiety and depression. Returning to normal family, social and work life can be difficult. Artificial eyes have been hand-painted at the National Artificial Eye Service since 1948. Each eye takes over six weeks to make. Technology advances mean alternatives are now possible. A digital photograph of the unaffected eye printed onto specialised paper and pressed onto the artificial eye is simpler and faster. Although the printed eye appears more life-like, there is no research evidence to show whether patients prefer it, the process can be done on a larger scale or save the National Health Service (NHS) money.

### Design and methods

Thirty-five adults will be invited to participate. A computer will randomly select which artificial eye type (hand-painted or printed) they will receive first. After wearing the first eye for two weeks, they will wear the other type. Using questionnaires before and after wearing each eye, we will ask patients how satisfied they were with each eye, and how it affected their confidence and well-being. We will also interview some patients and family members to get a better understanding of their thoughts. We will also measure eye production times and costs.

### Dissemination

Results will be presented at ophthalmology conferences. Study findings will be made available to participants, patient groups, NHS commissioners and reported on relevant patient-focused websites.

## *REVISED* Amendments from Version 1

We have revised the manuscript in light of the comments received from Reviewer 1, which mainly has resulted in additional information being added in line with the SPIRIT checklist. We have:

- Added confirmation of 'prospective' registration;
- Clarified medical device status;
- Addressed the query regarding the objective to 'identify a primary outcome measure';
- Stated the protocol version, uploaded it to OSF and included the sponsor details.
- Added information regarding intervention adherence strategies and concomitant care;
- Clarified which artificial eye participants wear while the second one is being manufactured, and whether they are able to retain the artificial eyes manufactured for them.
- Added further information about the data being collected, allocation sequence, masking, and confirmed that there would be no interim analyses or stopping rules.
- Added details on qualitative interview consent, auditing, and data sharing.

**Any further responses from the reviewers can be found at the end of the article**

## Open Peer Review

**Current Peer Review Status:**

**Version 2**

Reviewer Report 22 March 2023

https://doi.org/10.3310/nihropenres.14534.r29107

**© 2023 Cotton S.** This is an open access peer review report distributed under the terms of the Creative Commons Attribution License, which permits unrestricted use, distribution, and reproduction in any medium, provided the original work is properly cited.

**Seonaidh Cotton** http://orcid.org/0000-0002-7883-0608

Health Services Research Unit, University of Aberdeen, Aberdeen, UK

Many thanks for giving me the opportunity to assess the revisions to this article. The authors have addressed all the comments I had raised previously, and I am happy to update the status to Approved.

***Reviewer Expertise:*** Trialist, trial manager

**I confirm that I have read this submission and believe that I have an appropriate level of expertise to confirm that it is of an acceptable scientific standard.**

**Version 1**

Reviewer Report 13 February 2023

https://doi.org/10.3310/nihropenres.14436.r28963

**© 2023 Karlin J.** This is an open access peer review report distributed under the terms of the Creative Commons Attribution License, which permits unrestricted use, distribution, and reproduction in any medium, provided the original work is properly cited.

**Justin Karlin**

Division of Orbital and Ophthalmic Plastic Surgery, Doheny and Stein Eye Institutes, University of California, Los Angeles, Los Angeles, CA, USA

The authors have designed a crossover clinical trial to assess the feasibility and cost of using digitally printed (versus hand painted) ocular prostheses. Their inclusion and exclusion criteria, randomization and crossover plan, outcome measures (e.g. delivery time and patient satisfaction), data collection plan, and statistical analyses are clearly delineated. Written informed consent will be obtained from participants and there is minimal risk of harm. In summary, the design of the study is appropriate to answer the questions posed, and the information provided will certainly be valuable in designing a future sufficiently powered randomized controlled clinical trial.

**Is the rationale for, and objectives of, the study clearly described?**

Yes

**Is the study design appropriate for the research question?**

Yes

**Are sufficient details of the methods provided to allow replication by others?**

Yes

**Are the datasets clearly presented in a useable and accessible format?**

Yes

***Reviewer Expertise:*** I am an orbital and ophthalmic plastic surgeon. I have taken care of at least 50 patients with anophthalmic sockets.

**I confirm that I have read this submission and believe that I have an appropriate level of expertise to confirm that it is of an acceptable scientific standard.**

Author Response 03 Mar 2023

**Judith Watson**

We thank you taking the time to review our paper and for your positive comments on our paper.

Reviewer Report 23 December 2022

https://doi.org/10.3310/nihropenres.14436.r28899

**© 2022 Cotton S.** This is an open access peer review report distributed under the terms of the Creative Commons Attribution License, which permits unrestricted use, distribution, and reproduction in any medium, provided the original work is properly cited.

**Seonaidh Cotton** http://orcid.org/0000-0002-7883-0608

Health Services Research Unit, University of Aberdeen, Aberdeen, UK

This study has been funded, has received REC approvals and has completed recruitment. As such, this review does not comment on scientific value or methods employed within the trial, but focuses on the complete reporting of the protocol (with reference to the SPIRIT statement https://www.spirit-statement.org/). This is a really interesting study and I look forward to seeing results from it in the future.

There is no information in the manuscript as to whether the artificial eyes would be considered a medical device and/or are CE marked and it would be helpful to include this to satisfy readers that all necessary approvals were in place.

It is not clear how the objective to “identify a primary outcome measure” is being addressed within the current study – is this in relation to missing data, or is there work towards a core outcome set? The authors may wish to include more detail in the manuscript in this regard.

Was the trial prospectively registered (SPIRIT 2a)?

The version of the protocol is not referenced in the manuscript (SPIRIT 3). Could the full trial protocol be included in the OSF documentation link (SPIRIT 31c)?

The name and contact information for the trial sponsor is not included in the manuscript (SPIRIT 5b), and their role in the study is not provided (SPIRIT 5c).

Criteria for discontinuing the intervention (SPIRIT 11b), strategies to improve adherence to intervention protocols (SPIRIT 11c) and relevant concomitant care/interventions permitted/prohibited (SPIRIT 11d) are not explicitly provided in the manuscript. I wasn't clear what happened to participants between clinic 3 and clinic 4 – did they continue to wear the first eye while the second one was manufactured (and could this have introduced any bias)?

Given the patient pathway includes advertising the study on websites, how was the number/proportion of eligible patients defined (and what was used as the denominator) (SPIRIT 12)

Consideration could be given to including bit more information about allocation to fully cover the items on the SPIRIT checklist – sequence generation (SPIRIT 16a), allocation concealment mechanism (SPIRIT 16b) and implementation (SPIRIT 16c). There was no masking – are the digitally printed and hand painted artificial eyes easily identifiable visually (and if not, could participants and their close contact have been masked)?

The SPIRIT guidance on data management (SPIRIT 19) suggests information could be included on data entry and any processes to promote data quality, which are not currently included in the manuscript.

Given this is a feasibility trial, I presume that there will be no interim analysis or stopping guidelines, but this could be stated in the manuscript (SPIRIT 21b).

Were there plans to audit trial conduct (SPIRIT 23)?

Have there been any protocol modifications since the start of the study (SPIRIT 25)?

How (and by whom) is consent for the qualitative interviews sought? Is this consent recorded in writing or verbally as part of the interview? (SPIRIT 26b)

Who will have access to the final trial data set (SPIRIT 29)? Are there plans for sharing the dataset or statistical code (SPIRIT 31c)?

What are the provisions for ancillary and post-trial care (SPIRIT 30) - for example could participants chose to retain one of the artificial eyes?

**Is the rationale for, and objectives of, the study clearly described?**

Yes

**Is the study design appropriate for the research question?**

Yes

**Are sufficient details of the methods provided to allow replication by others?**

Partly

**Are the datasets clearly presented in a useable and accessible format?**

Not applicable

***Reviewer Expertise:*** Trialist, trial manager

**I confirm that I have read this submission and believe that I have an appropriate level of expertise to confirm that it is of an acceptable scientific standard, however I have significant reservations, as outlined above.**

## Author Response 03 Mar 2023

**Judith Watson**

Thank you for your very helpful comments and have made the necessary changes as detailed below:

There is no information in the manuscript as to whether the artificial eyes would be considered a medical device and/or are CE marked and it would be helpful to include this to satisfy readers that all necessary approvals were in place.

*Response: We have added clarification to the manuscript, stating that artificial eyes are classed by the Medicines and Healthcare products Regulatory Agency as "custom-made medical devices" intended for the sole use of a particular patient. Products manufactured in-house in a healthcare establishment that is conducting the study are not subject to the provisions of UK*

*Medical Devices Regulations 2002, provided that the device is being manufactured and used on patients within the sole legal entity. Since there is no objective/intention to place the device on the market currently, and we manufacture the device in-house for our own patients within Leeds Teaching Hospitals NHS Trust, in the context of this study, does not constitute a medical devices trial requiring MHRA notification. MHRA approval would be sought if the product is to be placed on the market in the future or a multi-centre trial was initiated.*

It is not clear how the objective to “identify a primary outcome measure” is being addressed within the current study – is this in relation to missing data, or is there work towards a core outcome set? The authors may wish to include more detail in the manuscript in this regard.

*Response: Thank you for this comment. We have now added some detail to the relevant objective in the manuscript*.

Was the trial prospectively registered (SPIRIT 2a)?

*Response: The text ‘prospectively registered on…’has been added to the date given*.

The version of the protocol is not referenced in the manuscript (SPIRIT 3). Could the full trial protocol be included in the OSF documentation link (SPIRIT 31c)?

*Response: The protocol version has been added to the Ethics and Oversight Arrangement sections as well as being uploaded to OSF*.

The name and contact information for the trial sponsor is not included in the manuscript (SPIRIT 5b), and their role in the study is not provided (SPIRIT 5c).

*Response: We have now added the name, contact details and role of the Sponsor (Acknowledgements section)*.

Criteria for discontinuing the intervention (SPIRIT 11b, strategies to improve adherence to intervention protocols (SPIRIT 11c) and relevant concomitant care/interventions permitted/prohibited (SPIRIT 11d) are not explicitly provided in the manuscript.

*Response: Thank you for pointing out these omissions. We have now added relevant information relating to these points*.

I wasn't clear what happened to participants between clinic 3 and clinic 4 – did they continue to wear the first eye while the second one was manufactured (and could this have introduced any bias)?

*Response: Correct, participants wore the first eye they were provided with until the second one was manufactured and fitted. Or if they preferred, they could revert back to their original artificial eye in the meantime*.

Given the patient pathway includes advertising the study on websites, how was the number/proportion of eligible patients defined (and what was used as the denominator)

(SPIRIT 12).

*Response: We have edited the text (Feasibility data) to make this clearer*.

Consideration could be given to including bit more information about allocation to fully cover the items on the SPIRIT checklist – sequence generation (SPIRIT 16a), allocation concealment mechanism (SPIRIT 16b) and implementation (SPIRIT 16c).

*Response: Thank you for pointing this out. We have now added the additional information*.

There was no masking – are the digitally printed and hand painted artificial eyes easily identifiable visually (and if not, could participants and their close contact have been masked)?

*Response: Yes they are identifiable when not wearing, so masking was not possible as noted in ‘Level of masking’*.

The SPIRIT guidance on data management (SPIRIT 19) suggests information could be included on data entry and any processes to promote data quality, which are not currently included in the manuscript.

*Response: We have now added this information*.

Given this is a feasibility trial, I presume that there will be no interim analysis or stopping guidelines, but this could be stated in the manuscript (SPIRIT 21b).

*Response: This is correct and as suggested, we have added this to the manuscript*.

Were there plans to audit trial conduct (SPIRIT 23)?

*Response: We have added details about the planned activities*.

Have there been any protocol modifications since the start of the study (SPIRIT 25)?

*Response: No protocol modifications have been made since regulatory approvals were obtained and this is now stated in the manuscript*.

How (and by whom) is consent for the qualitative interviews sought? Is this consent recorded in writing or verbally as part of the interview? (SPIRIT 26b)

*Response: The study consent form contained statements related to the qualitative interviews*.

*Those interviewed had their consent re-confirmed verbally prior to the interview taking place. This detail has been added to the manuscript*.

Who will have access to the final trial data set (SPIRIT 29)? Are there plans for sharing the dataset or statistical code (SPIRIT 31c)?

*Response: Additional information has been added to the Data Availability section regarding this*.

What are the provisions for ancillary and post-trial care (SPIRIT 30) - for example could participants chose to retain one of the artificial eyes?

*Response: Participants are allowed to retain the eyes made during the study. Text has been added to confirm this*.
